# Does *KRAS* Testing in Metastatic Colorectal Cancer Impact Overall Survival? A Comparative Effectiveness Study in a Population-Based Sample

**DOI:** 10.1371/journal.pone.0094977

**Published:** 2014-05-01

**Authors:** Heather Spencer Feigelson, Chan Zeng, Pamala A. Pawloski, Adedayo A. Onitilo, C. Sue Richards, Monique A. Johnson, Tia L. Kauffman, Jennifer Webster, Carsie Nyirenda, Gwen L. Alexander, Clara Hwang, Deanna Cross, Catherine A. McCarty, Robert L. Davis, Denise Schwarzkopf, Andrew E. Williams, Stacey Honda, Yihe Daida, Lawrence H. Kushi, Thomas Delate, Katrina A. B. Goddard, on behalf of the CERGEN Study Team

**Affiliations:** 1 Institute for Health Research, Kaiser Permanente Colorado, Denver, Colorado, United States of America; 2 HealthPartners Institute for Education and Research, Bloomington, Minnesota, United States of America; 3 Department of Hematology/Oncology, Marshfield Clinic Weston Center, Weston, Wisconsin, and Marshfield Clinic Research Foundation, Marshfield, Wisconsin, United States of America; Clinical Epidemiology Unit, School of Population Health, University of Queensland, Brisbane, Queensland, Australia; 4 Molecular and Medical Genetics, Oregon Health & Science University, Portland, Oregon, United States of America; 5 The Center for Health Research Kaiser Permanente Northwest, Portland, Oregon, United States of America; 6 Henry Ford Health System, Department of Public Health Sciences, Detroit, Michigan, United States of America; 7 Henry Ford Health System, Department of Internal Medicine, Division of Hematology/Oncology, Detroit, Michigan, United States of America; 8 Marshfield Clinic Research Foundation, Marshfield, Wisconsin, United States of America; 9 Essentia Institute of Rural Health, Duluth, Minnesota, United States of America; 10 Department of Pediatrics, University of Tennessee Health Sciences Center, Memphis, Tennessee, United States of America; 11 Center for Health Research, Kaiser Permanente Hawai’i, Honolulu, Hawaii, United States of America; 12 Division of Research, Kaiser Permanente Northern California, Oakland, California, United States of America; 13 Kaiser Permanente Colorado, Pharmacy Department, Denver, Colorado, United States of America; The Chinese University of Hong Kong, Hong Kong

## Abstract

**Purpose:**

Epidermal growth factor receptor (EGFR) inhibitors are approved for treating metastatic colorectal cancer (CRC); *KRAS* mutation testing is recommended prior to treatment. We conducted a non-inferiority analysis to examine whether *KRAS* testing has impacted survival in CRC patients.

**Patients and Methods:**

We included 1186 metastatic CRC cases from seven health plans. A cutpoint of July, 2008, was used to define two *KRAS* testing time period groups: “pre-testing” (n = 760 cases) and “post-testing” (n = 426 cases). Overall survival (OS) was estimated, and the difference in median OS between the groups was calculated. The lower bound of the one-sided 95% confidence interval (CI) for the difference in survival was used to test the null hypothesis of post-testing inferiority. Multivariable Cox regression models were constructed to adjust for covariates.

**Results:**

The median unadjusted OS was 15.4 months (95% CI: 14.0–17.5) and 12.8 months (95% CI: 10.0–15.2) in the pre- and post-testing groups, respectively. The OS difference was −2.6 months with one-sided 95% lower confidence bound of −5.13 months, which was less than the non-inferiority margin (−5.0 months, unadjusted p = 0.06), leading to a failure to reject inferiority of OS in the post-testing period. In contrast, in the adjusted analysis, OS non-inferiority was identified in the post-testing period (p = 0.001). Sensitivity analyses using cutpoints before and after July, 2008, also met the criteria for non-inferiority.

**Conclusion:**

Implementation of *KRAS* testing did not influence CRC OS. Our data support the use of *KRAS* testing to guide administration of EGFR inhibitors for treatment of metastatic CRC without diminished OS.

## Introduction

While survival rates in individuals with colorectal cancer (CRC) have increased significantly in recent years, survival among patients with metastatic CRC remains poor, with five-year survival of just 12% [1]. Cetuximab and panitumumab are monoclonal antibodies approved for the treatment of refractory CRC that block the epidermal growth factor receptor (EGFR) signaling pathway in tumor cells and, thus, can slow tumor progression [2]. However, retrospective re-analysis of clinical trial data demonstrated that these drugs do not benefit patients whose tumors harbor a *KRAS* mutation [3], [4]. In April 2009, the American Society of Clinical Oncology (ASCO) recommended that patients with metastatic CRC who are candidates for EGFR inhibitors have their tumor tested for *KRAS* mutations, and that those with a *KRAS* mutation in codon 12 or 13 not receive anti-EGFR treatment [3]. Shortly thereafter, the FDA recommended re-labeling of EGFR inhibitors to refer to *KRAS* testing [5]. The impact of this is not insignificant, as up to 40% of CRC tumors harbor a *KRAS* mutation [6]–[8]. For patients with these mutations, an alternative targeted therapy does not currently exist.

We have previously documented the rapid diffusion of this technology into clinical practice by identifying a pronounced increase in *KRAS* testing beginning only one month after clinical trials were presented at the ASCO national conference in June, 2008 [9]. Despite clear recommendations for, and rapid uptake of, *KRAS* testing, complexities in *KRAS* testing and subsequent treatment decisions remain. For example, recent data have shown that patients with CRC tumors harboring the *KRAS* p.G13D mutation may derive some benefit from cetuximab treatment; although, not as much as those with *KRAS* wild type tumors [10]. Additionally, *KRAS* mutations are not limited to codons 12 and 13. Mutations in *KRAS* exon 4 were found to occur commonly and have been associated with more favorable clinical outcomes than other mutations [11]. Nevertheless, the effectiveness of EGFR inhibitors in patients with less common *KRAS* mutations remains unknown.

With the uncertainty around the significance of specific *KRAS* mutations, and with no proven alternative treatment for those who have tumors with *KRAS* mutations, we sought to determine whether *KRAS* testing has impacted survival in metastatic CRC patients.

## Materials and Methods

### Study Population

We conducted a non-inferiority study including patients from seven sites in the Cancer Research Network (CRN), a consortium of non-profit research centers based in integrated healthcare delivery organizations [12]. This study included nearly all (>90%) metastatic CRC cases diagnosed at six CRN member institutions: Kaiser Permanente regions Northwest, Hawaii, and Colorado, Henry Ford Health System, Marshfield Clinic, and HealthPartners. Due to its large population, we included a 28% random sample of eligible cases diagnosed at Kaiser Permanente Northern California.

This study was approved by the Institutional Review Boards (IRB) at Kaiser Permanente Northwest, Kaiser Permanente Hawaii, Kaiser Permanente Colorado, Marshfield Clinic Research Foundation, and Henry Ford Health System. The IRBs for the remaining sites ceded authority to the Kaiser Permanente Northwest IRB. The IRBs waived the need for written informed consent from the participants. Criteria for waiver of written informed consent included minimal risk study and retrospective review of data already in existence. A small number (<1%) of health plan members have elected not to participate in unconsented research protocols and were excluded.

We identified cases aged 18 and older with International Classification of Diseases for Oncology codes C18.0, C18.2–C20.9 and histology codes 8000 − <8500. We included all cases of stage IV CRC diagnosed between January 1, 2006 and December 31, 2009, and cases with an initial diagnosis of stage III CRC diagnosed between January 1, 2004, and December 31, 2008, who progressed to distant metastatic CRC (determined using chart review). Distant metastatic CRC was defined as metastases to distant lymph nodes, brain, lung, liver, peritoneum, or other distant organs. Cases with metastases only to regional lymph nodes were excluded.

We also applied enrollment or encounter-based criteria to ensure adequate information was available on follow-up and treatment. Eligible cases were enrolled for at least one year after diagnosis (allowing for up to 3-month gaps in enrollment), deceased within one year of diagnosis but enrolled at time of death, or with at least one medical encounter (of any type) between 7 and 12 months following initial diagnosis.

### Data Collection

We obtained data from each site’s Virtual Data Warehouse (VDW), which has been described elsewhere [13]. Each CRN site maintains a tumor registry where clinical data are abstracted from the medical chart into an electronic database and from where the VDW tumor registry file is populated. We used the VDW to identify eligible cases and obtain data on patient characteristics (gender, age at diagnosis, race, ethnicity, body mass index, smoking status, alcohol use, vital status, Medicare status, and comorbid conditions), tumor characteristics (cancer site, stage, histology), and treatment history (chemotherapy, immunotherapy, radiation therapy, surgery). As a measure of general health, the Quan comorbidity score was calculated. This score is an unweighted version of the Charlson Comorbidity Index containing 17 comorbidities captured within one year of CRC [14]. Trained abstractors at each site manually extracted additional information on each case using standard data collection forms. Abstracted information included family history of cancer, detailed treatment history, palliative care, genetic testing (including *KRAS*), and imaging to assess disease progression. Abstractors also verified eligibility, race, ethnicity, smoking, and alcohol use.

### *KRAS* Genetic Testing

We included analysis of *KRAS* codons 12 and 13 only. When available, we abstracted *KRAS* results from testing ordered as part of clinical care (n = 428) from commercial or academic-based laboratories. For patients who did not receive clinical *KRAS* testing, archived pathology specimens (formalin fixed, paraffin embedded slides) were obtained, when possible, and *KRAS* genotyping was performed as part of the research study in a clinical diagnostic laboratory at the Oregon Health & Sciences University (OHSU). At OHSU, manual microdissection of the tumor tissue was performed and following DNA extraction, the regions of interest were amplified. Mutation detection was performed using standard bidirectional sequencing on an ABI 3100. A *KRAS* mutation, if present, had to be evident in both forward and reverse reactions. All samples were tested in duplicate (two forward and two reverse reactions). As we planned to combine *KRAS* results from multiple laboratories employing different mutation detection methods, we conducted a validation study to determine *KRAS* test reproducibility and found 90% concordance in test results across the laboratories [15].

### Statistical Analysis

We defined the “pre-testing group” as cases diagnosed before July 1, 2008, and the “post-testing group” as cases diagnosed on or after July 1, 2008. We chose July 1, 2008, as our cutpoint for the testing periods because we documented a pronounced increase in *KRAS* testing beginning only one month after clinical trials were presented at the ASCO national conference in June, 2008 [9]. The outcome of interest was overall survival (OS), defined as the time from date of metastatic CRC diagnosis to date of death or date of last follow up (12/31/2011). For cases who presented with stage IV disease, the date of metastatic CRC was equal to their date of diagnosis. For stage III cases, date of metastatic CRC diagnosis was determined from chart review. All analyses were performed using the SAS statistical package version 9.2 (SAS Institute, Cary, NC).

The study was designed to assess the non-inferiority of OS in the post-testing group versus the pre-testing group. A non-inferiority test was the appropriate test for our study question, which was to determine whether the guidelines regarding *KRAS* testing in CRC patients has impacted survival. Because the testing recommendations state that EGFR inhibitors should not be used in the treatment of CRC tumors harboring *KRAS* mutations in codon 12 or 13, it was important to demonstrate that survival was no worse (i.e., “not inferior”) in the post-testing era than in the pre-testing era. OS was estimated using the Kaplan-Meier method. The difference in median OS between the two groups was calculated, and the lower bound of the one-sided 95% confidence interval (CI) of this difference was used to test the null hypothesis of inferiority of the post-testing group against the alternative hypothesis of non-inferiority [16]. The test statistics was calculated as:
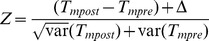
Were *T_mpost_* = median OS in post-testing, *T_mpre_* = median OS in pre-testing group and Δ = predefined inferiority margin (−5 months). This test statistic is assumed to have a standard normal distribution, which is used to calculate the p-value.

Based on previous studies [4], [17]–[19], we assumed a median OS of 20 months among cases in the pre-testing group, and set the non-inferiority margin at −5.0 months. Three studies that had evaluated survival by *KRAS* mutation status and EGFR inhibitor use found that among patients receiving EGFR inhibitors, the survival difference comparing those *KRAS* wild-type tumors versus *KRAS* mutated tumors was 7.4 months [19], 3.2 months [17], and 5.0 months [18]. We chose 5.0 months since it represented the median of these results. Thus, the null hypothesis would be rejected if the lower confidence bound was greater than −5.0 months, indicating survival was not worse in the post-testing group than in the pre-testing group. With a 1-sided alpha level of 5% and a power of 90%, a minimum of 400 cases per group would be required to demonstrate non-inferiority.

Patient characteristics, tumor characteristics and treatment history were compared between cases in the pre-testing and post-testing groups using the chi-square test for categorical variables and two sample t-tests or Wilcoxon rank sum tests for continuous variables.

After conducting our un-adjusted non-inferiority tests, we used a multivariable Cox proportional hazards model to account for covariates that may also influence overall survival. The proportional hazards assumption for the effect of the post-testing versus pre-testing groups was examined using Schoenfeld residuals. A Cox regression model was constructed, adjusting for patient characteristics (gender, age, race, ethnicity, smoking status, presence of comorbidities and study site), stage at diagnosis, and treatment history (receipt of surgery, radiation, and/or chemotherapy). We created three mutually exclusive categories to define chemotherapy use for treatment of metastatic disease: EGFR inhibitor use (with our without other chemotherapy), chemotherapy without the addition of EGFR inhibitor, or no chemotherapy. Under the proportional hazards assumption, the non-inferiority margin of −5.0 months of survival time would correspond to a hazard ratio (HR) of 1.33. Thus, if the one-sided 95% upper confidence bound of the estimated HR from the Cox model was less than 1.33, we would conclude that the survival time in the post-testing period was not inferior to the survival time in the pre-testing period.

Because the July 1, 2008, cutpoint is only an estimate of when *KRAS* testing was implemented, we also examined non-inferiority results using cutpoints April 1, June 1, August 1, and October 1, 2008, to assess whether our results were sensitive to our choice of cutpoint date.

## Results

A total of 1186 metastatic CRC cases were included, 760 (64%) in the pre-testing group and 426 (36%) in the post-testing group. We included 922 (78%) cases diagnosed at stage IV CRC and 264 (22%) cases diagnosed at stage III CRC who developed distant metastatic disease. The median follow-up time was 13.9 months (range 0–71 months). Table 1 displays patient characteristics for the pre- and post- testing groups. The post-testing group was slightly, but not statistically significantly, younger (mean age 65.5 versus 66.9, p = 0.10), included more Non-Hispanic whites (66.9% versus 62.6%), fewer African Americans (8.7% versus 11.8%), and more nonsmokers (48.6% versus 41.8%). The post-testing group had fewer samples that were insufficient for *KRAS* testing (4.0% versus 8.2%), more unavailable samples (25.8% versus 20.8%) and more patients with *KRAS* testing ordered as part of their clinical care (40.1% versus 33.8%).

**Table 1:** Characteristics of 1,186 patients diagnosed with metastatic colorectal cancer by group (diagnosis before or after July 1, 2008).

	Total	Pre-testing	Post-testing	
	N = 1186 (%)	N = 760 (%)	N = 426 (%)	p-value
Gender				
Male	601 (50.7)	394 (51.8)	207 (48.7)	0.30
Female	584 (49.3)	366 (48.2)	218 (51.3)	
Mean (± SD) age at diagnosis	66.4±13.5	66.9±13.0	65.5±14.2	0.10
Study site				
1	425 (35.8)	254 (33.4)	171 (40.1)	0.45
2	124 (10.5)	85 (11.2)	39 (9.2)	
3	100 (8.4)	67 (8.8)	33 (7.7)	
4	193 (16.3)	127 (16.7)	66 (15.5)	
5	62 (5.2)	40 (5.3)	22 (5.2)	
6	133 (11.2)	88 (11.6)	45 (10.6)	
7	149 (12.6)	99 (13.0)	50 (11.7)	
Race/ethnicity				
Non-Hispanic White	761 (64.2)	476 (62.6)	285 (66.9)	0.02
African-American	127 (10.7)	90 (11.8)	37 (8.7)	
Asian/Pacific Islander	118 (9.9)	70 (9.2)	48 (11.3)	
Hispanic	69 (5.8)	40 (5.3)	29 (6.8)	
Other/Unknown	111 (9.4)	84 (11.1)	27 (6.3)	
Smoking Status				
Current Smoker	136 (11.5)	82 (10.8)	54 (12.7)	0.01
Ex-Smoker	467 (39.4)	315 (41.4)	152 (35.7)	
Nonsmoker	525 (44.3)	318 (41.8)	207 (48.6)	
Unknown	58 (4.9)	45 (5.9)	13 (3.1)	
Quan comorbidity score				
Mean ± SD	3.43±2.96	3.46±2.89	3.39±3.08	0.27
Cancer stage				
Stage III with progression	264 (22.3)	170 (22.4)	94 (22.1)	0.90
Stage IV	922 (77.7)	590 (77.6)	332 (77.9)	
KRAS test result				
Mutation	326 (27.5)	212 (27.9)	114 (26.8)	0.02
Wild Type	513 (43.3)	328 (43.2)	185 (43.4)	
Insufficient	79 (6.7)	62 (8.2)	17 (4.0)	
Sample unavailable	268 (22.6)	158 (20.8)	110 (25.8)	
KRAS test ordered for clinical care				
No	758 (63.9)	503 (66.2)	255 (59.9)	0.03
Yes	428 (36.1)	257 (33.8)	171 (40.1)	

Table 2 shows the treatment patterns in the pre- and post-testing groups for stage III CRC. The majority of cases had surgery (92.9%) and chemotherapy (80.5%) at initial diagnosis. After distant metastases were diagnosed, fewer cases had chemotherapy in the post-testing group (66.3% versus 72.9%, p = 0.26), but this difference was not statistically significant. Specifically, the number of lines of treatment for metastatic disease was reduced in the post-testing group (1.31 versus 2.04, p = 0.01), as was the use of EGFR inhibitors (17.0% versus 30.6%).

**Table 2:** Treatment patterns for 264 metastatic colorectal cancer patients diagnosed at Stage III by group.

	Total	Pre-testing	Post-testing	
	N = 264 (%)	N = 170 (%)	N = 94 (%)	p-value
Surgery for initial diagnosis (% Yes)	224 (92.9)	142 (92.8)	82 (93.2)	0.91
Radiation for initial diagnosis (% Yes)	60 (25.0)	37 (24.3)	23 (26.1)	0.76
Chemotherapy for initial diagnosis (% Yes)	194 (80.5)	121 (79.1)	73 (83.0)	0.47
Chemotherapy for metastatic diagnosis (% Yes)	185 (70.6)	124 (72.9)	61 (66.3)	0.26
Number of lines of therapy for treatment of initial diagnosis				
Mean ± SD	1.10±0.88	1.05±0.88	1.18±0.89	0.23
Number of lines of therapy for treatment of metastatic diagnosis				
Mean ± SD	1.78±1.79	2.04±1.99	1.31±1.23	0.01
EGFR inhibitor use for metastatic diagnosis				
EGFR inhibitor^1^	68 (25.8)	52 (30.6)	16 (17.0)	0.05
Chemotherapy without EGFR	122 (46.2)	73 (42.9)	49 (52.1)	
No Chemotherapy	74 (28.0)	45 (26.5)	29 (30.9)	

1 EGFR inhibitor use alone or in combination with other chemotherapy.

Unlike stage III cases, only half (52.9%) of stage IV patients received surgery (Table 3), and fewer in the post-testing group than in the pre-testing group (45.0% versus 57.3%, p<0.001). Nearly 70% received some chemotherapy. The post-testing group had fewer lines of treatment (1.48 versus 1.93, p = 0.004) and a 50% reduction in EGFR inhibitor use (12.0% versus 24.6%).

**Table 3:** Treatment patterns for 922 metastatic colorectal cancer patients diagnosed at Stage IV by group.

	Total	Pre-testing	Post-testing	p-value
	N = 922 (%)	N = 590 (%)	N = 332 (%)	
Surgery for initial diagnosis (% Yes)	486 (52.9)	337 (57.3%)	149 (45.0)	<0.001
Radiation for initial diagnosis (% Yes)	143 (15.5)	96 (16.3%)	47 (14.2)	0.40
Chemotherapy for initial diagnosis (% Yes)	625 (68.6)	409 (69.9)	216 (66.3)	0.25
Total lines of chemotherapy				
Mean ± SD	1.77±1.81	1.93±1.98	1.48±1.43	0.004
EGFR inhibitor use				
EGFR inhibitor^1^	185 (20.1)	145 (24.6)	40 (12.0)	<0.001
Chemotherapy without EGFR	445 (48.3)	265 (44.9)	180 (54.2)	
No Chemotherapy	292 (31.7)	180 (30.5)	112 (33.7)	

1 EGFR inhibitor use alone or in combination with other chemotherapy.

**Figure 1:**
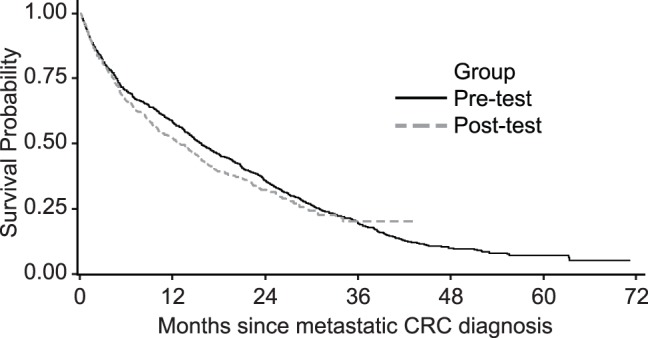
Overall survival in the pre-testing (solid line) and post-testing (dashed line) groups.

Kaplan-Meier curves of OS for the pre- and post-testing groups show similar survival for the two groups (Figure 1). The median OS was 15.4 months (95% CI: 14.0–17.5) in the pre-testing group and 12.8 months (95% CI: 10.0–15.2) in the post-testing group. The difference in OS between post-testing group and pre-testing was −2.6 months with a one-sided 95% lower confidence bound of −5.13 months. Because the lower confidence bound is less than −5.0 months, we cannot reject the null hypothesis of inferiority (p = 0.06).

In the multivariable Cox regression model (Table 4), age at diagnosis (p = 0.02), race/ethnicity (p = 0.01), stage (p = <0.0001), comorbidity score (p = 0.002) and treatment (surgery and chemotherapy, p = <0.0001) were all significant predictors of survival. However, the upper confidence bound for the HR comparing the post- versus pre- testing groups was 1.19, which is less than the HR margin of 1.33. Thus, survival is not inferior in the post-testing period when other predictors have been taken into account.

**Table 4:** Hazard ratios for overall survival by testing group and other characteristics, estimated from multivariable cox proportional hazards regression.

Variable	HR (95% CI)^1^	P-value
Group		
Pre-testing	1.0	
Post-testing	1.05 (1.19)^2^	<0.001
Age at diagnosis (years)	1.007 (1.00, 1.01)	0.02
Gender		0.54
Female	1.0	
Male	1.04 (0.91, 1.20)	
Race/ethnicity		0.01
Non-Hispanic white	1.0	
African-American	1.10(0.86, 1.42)	
Asian/Pacific Islander	0.83 (0.63, 1.09)	
Hispanic	0.80 (0.59, 1.08)	
Other/Unknown	1.37 (1.09, 1.73)	
Smoking status		0.05
Nonsmoker\Never Smoker	1.0	
Ex-Smoker	1.18 (1.02, 1.31)	
Current Smoker	1.21 (0.98, 1.50)	
Cancer stage		<0.0001
Stage III with progression	0.61 (0.51, 0.72)	
Stage IV	1.0	
Quan comorbidity score	1.04 (1.01, 1.06)	0.002
Surgery for metastatic diagnosis		<0.0001
No	1.0	
Yes	0.51 (0.44, 0.58)	
Radiation for metastatic diagnosis		0.56
No	1.0	
Yes	0.95 (0.79, 1.14)	
Chemotherapy for metastatic diagnosis		<0.0001
No Chemotherapy	1.0	
Chemo without EGFR	0.23 (0.19, 0.28)	
EGFR Inhibitor	0.24 (0.19, 0.29)	
Sites		0.06
1	1.0	
2	0.80 (0.63, 1.02)	
3	1.15 (0.94, 1.41)	
4	1.30 (0.95, 1.79)	
5	1.02 (0.78, 1.33)	
6	1.09 (0.81, 1.47)	
7	0.91 (0.72, 1.14)	

1 Two-sided 95% CI from the multivariable Cox model.

2 One-sided 95% upper confidence bound for post- versus pre-testing group; corresponding p-value <0.001, which rejects the inferiority null hypothesis that OS is worse in the post-testing group than in the pre-testing group.

Because the cutpoint of July 1, 2008, is only an approximation of when *KRAS* testing was widely implemented, we estimated OS and compared median survival for the pre- and post-testing groups using four other cutpoints: April 1, June 1, August 1, October 1, 2008 (Table 5). All of these dates meet the criteria for non-inferiority (i.e., the lower bound of the confidence limit was greater than −5.0, and the p-value testing OS was <0.05).

**Table 5:** Sensitivity analysis showing unadjusted non-inferiority test results for overall survival (OS) using different dates to specify pre- and post-testing groups for adoption of KRAS testing.

	Median overall survival (months)				
Cutpoint		Pre-testing	Post-testing	Difference, months (LCB)^1^	p value^2^
4/1/2008		14.94	14.02	−0.92 (−3.15)	0.001
6/1/2008		15.21	13.72	−1.49 (−3.87)	0.008
7/1/2008		15.44	12.83	−2.61 (−5.13)	0.059
8/1/2008		15.34	12.96	−2.38 (−4.96)	0.048
10/1/2008		14.88	13.92	−0.96 (−3.69)	0.008

1 Difference in months = Post-testing - Pre-testing; CB: one sided 95% lower confidence bound (LCB). If LCB>−5.0 months, then the post-testing OS is not worse than the pre-testing OS.

2 Unadjusted p-value for testing inferiority of OS in post- vs. pre-testing group.

**Figure 2:**
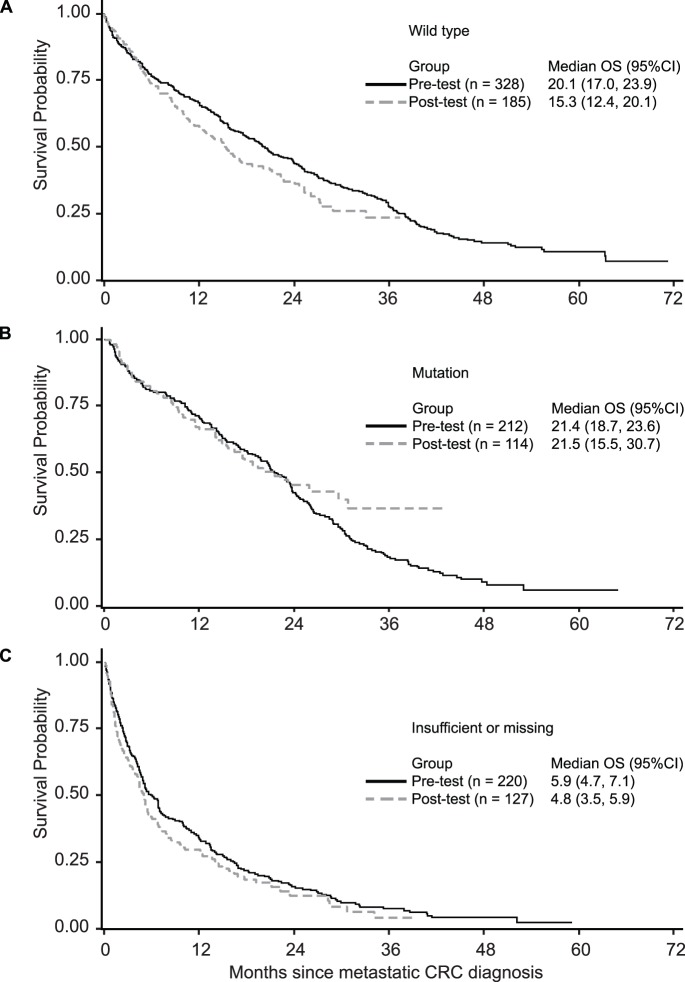
Overall survival in the pre-testing and post-testing groups by *KRAS* test result. **A.** Overall survival in the pre-testing and post-testing groups among those with *KRAS* wild-type tumors. **B.** Overall survival in the pre-testing and post-testing groups among those with *KRAS* mutations. **C.** Overall survival in the pre-testing and post-testing groups among those with no *KRAS* test results (as a result of insufficient or no tissue available for testing).

We also examined survival separately for patients with *KRAS* wild-type tumors, *KRAS* mutated tumors, and missing or insufficient tumor tissue to assess whether survival differed between the testing periods for any sub-group. Kaplan-Meier curves of OS by testing group and *KRAS* mutation status are shown in Figure 2. Median OS time was shorter among *KRAS* wild type patients in the post-testing group (15.3 months) compared to patients in the pre-testing group (20.1 months), and also compared to patients with *KRAS* mutations in either pre- or post- testing groups (21.4 and 21.5 months, respectively). This difference in survival is driven by data from one of the participating sites. We examined patient factors, such as age and stage at diagnosis, and treatment type (receipt of surgery, chemotherapy and radiation) and did not detect differences that might explain this observation (data not shown). Patients with missing or insufficient tumor samples were older, had more co-morbidities, and were less likely to receive surgical treatment or chemotherapy (data not shown), which is reflected in their short survival time (5.9 and 4.8 months) (Figure 2C).

## Discussion

*KRAS* testing in metastatic CRC patients prior to the administration of EGFR inhibitors has been rapidly integrated into oncology practice [9]. Our results demonstrate that the survival time for metastatic CRC patients diagnosed after *KRAS* testing had been recommended was not inferior compared to survival time among patients diagnosed before KRAS testing was recommended. This is especially notable because there is currently no alternative targeted treatment for patients with *KRAS* mutations that render EGFR inhibitors ineffective. Our multivariable analysis indicated that factors such as stage, treatment, presence of comorbidities, and age predictably influence survival, but being diagnosed in the recent era of *KRAS* testing did not.

*KRAS* testing recommendations were established based on evidence gathered retrospectively from randomized clinical trials [3], [4]. Our data, drawn from seven large integrated health plans across the U.S., offer some important advantages over data from clinical trials. First, our study is more representative of the spectrum of metastatic CRC patients; our population is ethnically diverse and has a broad age range (19–97 years). Second, we made no exclusions for health status as would be done for a clinical trial. Third, *KRAS* testing was performed prospectively and was used to guide treatment decisions.

Another important difference between our population and that of clinical trials is that *KRAS* testing was not performed on all patients. *KRAS* testing was ordered for 33.8% of patients in the pre-testing group, and 40.1% of patients in the post-testing group. Although a higher proportion of patients received clinical testing in the post-testing period as would be expected, there may be several reasons to explain why this percentage isn’t higher. Some providers order *KRAS* testing at the time of diagnosis, while others wait until they are considering use of EGFR inhibitors. Our study may not have captured testing that occurred later in care. More importantly, patients diagnosed with widespread metastatic disease, or who were not candidates for surgical resection, often did not have tissue available for *KRAS* testing. These patients would not be represented in clinical trials, but make up 30% of our patient population.

It is reassuring that our results, in spite of these important differences, support previous findings that withholding EGFR inhibitors from patients with tumors that harbor *KRAS* mutations does not negatively impact OS. In fact, our median OS is favorable compared to data from clinical trials. Data from a systematic review of *KRAS* testing and response to treatment with EGFR inhibitors in patients with advanced colorectal cancer [4] reported that median OS was between 6.6 and 24.9 months for patients with wild-type *KRAS*, and 4.4 and 17.5 months for patients with mutated *KRAS*. Our median OS was between 15.3 and 21.5 months, depending on *KRAS* mutation status and pre- versus post-testing period.

Our population-based data also present challenges. First, because this study was conducted in a “usual care” setting, rather than being derived from a clinical trial, we observed a wide variety of treatment protocols. Although treatments were very similar between the pre-testing and post-testing groups, it is difficult to fully account for treatment variation in the analysis. Also, patient mix and treatment protocols differ across participating sites. Controlling for site in our multivariable analysis may not completely account for these differences. Second, we chose the cutpoint of July, 2008, to reflect the presentation of data supporting *KRAS* testing at the ASCO meeting rather than a time point following formal recommendations or FDA labeling changes, because the latter dates lagged behind actual practice changes. It is likely that cases on either side of this cutpoint were misclassified; for example, *KRAS* testing may have been used to guide treatment for some patients in our “pre-testing” group. Further, it is likely that *KRAS* testing around this cutpoint was differentially applied by the clinical community to patients at various stages of treatment, disease progression, and health status. The results from our non-inferiority test provide evidence to support this. Non-inferiority tests are usually applied to clinical trial data [20], [21], where the process of randomization reduces the influence of bias, and where start dates and treatment are completely specified. In applying a non-inferiority test to observational data, bias can influence the test statistic. Our non-inferiority test results differed by the date we chose as a cutpoint. Specifically for the July 1, 2008, cutpoint, we could not conclude that the post-testing period survival was non-inferior to the pre-testing period survival. Using any of the other cutpoints we tested, we could conclude non-inferiority of survival in the post-testing period (i.e., survival was not worse in the post-testing group). Further, results of our multivariable model, adjusting for stage, treatment, comorbidity status, etc., supported non-inferiority.

In summary, our data support the use of *KRAS* testing to guide administration of EGFR inhibitors for treatment of metastatic CRC. To our knowledge, this is the first study to examine this question in a prospective, population-based study. *KRAS* testing recommendations were established based on retrospective analyses of clinical trials data, however, patient mix in the usual care setting is different than in the clinical trial setting, and outcomes may not be as favorable. Thus, it is reassuring that we find no negative impact on survival after the implementation of *KRAS* testing in community practice. *KRAS* testing has been rapidly integrated into oncology care [9], and is only one of many molecular markers currently being used to guide treatment decisions. It is important that we continue to evaluate the benefit of these molecular tools in both clinical trials and usual care settings to ensure optimal patient survival and quality of life.
